# BRUNNER SYNDROME: FROM GENETICS TO PSYCHIATRY - CASE REPORT

**DOI:** 10.1192/j.eurpsy.2023.1877

**Published:** 2023-07-19

**Authors:** A. L. Falcão, C. Oliveira, A. Lourenço, G. Soares, J. Petta

**Affiliations:** 1 Clínica 3, Centro Hospitalar Psiquiátrico de Lisboa, Lisboa, Portugal

## Abstract

**Introduction:**

Monoaminergic neurotransmitters affect motor control, emotions and cognitive functions. Their relative abundance depends on the action of monoamine oxidase A (MAOA) which catalyses their degradation into inactive metabolites. Brunner syndrome is a rare chromosome X-linked recessive inheritance genetic disorder, which causes loss of function of the MAOA gene, leading to a relative increase in monoamines in affected individuals (typically males). It manifests with moderate intellectual developmental disturbance associated with maladaptive externalising behaviours, low frustration tolerance and marked impulsivity.

**Objectives:**

To present a clinical case of patient diagnosed with Brunner Syndrome presenting with suicidal and homicidal ideation and to discuss it based on a non-systematic review of the literature.

**Methods:**

Case report and non-systematic review of the literature.

**Results: Case Report:**

Male born with neonatal asphyxia that consequently presented developmental retardation characterised by learning difficulties and emotional dysregulation. At 18 years of age, he began follow-up in Psychiatry due to self-injurious behaviours and several reactive suicide attempts (SA), being diagnosed with Intellectual Disability. At 22, he was hospitalised due to reactive depression, being discharged doing well with valproate, fluoxetine and olanzapine. Shortly after, he was arrested (aged 23-28) for attempted murder followed by SA. During imprisonment, he was diagnosed with Brunner’s Syndrome after his younger brother diagnosis. At 31, he was hospitalised for suicidal and homicidal ideation, revealing high impulsiveness and low frustration tolerance, and was discharged with compulsory psychiatric treatment. Three weeks later he was again hospitalised for symptoms recurrence having improved on Sertraline 50mg and Aripiprazole 400mg monthly.

**Discussion:**

Although scarce, the available evidence suggests that dietary modifications and the use of selective serotonin reuptake inhibitors may improve behaviour, reduce serotoninergic symptoms and possibly reverse some neurochemical abnormalities.

**Conclusions:**

Brunner syndrome is a rare genetic disorder with neuropsychiatric manifestations, whose course, treatment and prognosis are currently poorly known. Thus, the current treatment is empirical and further studies are needed.

**Disclosure of Interest:**

None Declared

